# Haplotype diversity and linkage disequilibrium at the *DRD2* locus among the tribes of western and southern regions of India

**DOI:** 10.4103/0971-6866.69327

**Published:** 2010

**Authors:** Aastha Aggarwal, Mansi Gauniyal, Ipsa Pattanayak, Gautam K. Kshatriya

**Affiliations:** Department of Anthropology, University of Delhi (North Campus), Delhi-110 007, India

**Keywords:** Ancestral haplotype, ethno-linguistic diversity, haplotype analysis, linkage disequilibrium, population structure

## Abstract

**BACKGROUND:**

Dopamine receptor D2 (DRD2) is an important gene having functional significance in the fields of neuropsychiatry and pharmacology and also has importance in evolutionary studies.

**MATERIALS AND METHODS:**

This study was undertaken to find out the haplotype distribution and linkage disequilibrium (LD) pattern for the three *TaqI* sites (*TaqI* ‘A’, *TaqI* ‘B’ and *TaqI* ‘D’) in the DRD2 gene in 232 unrelated individuals from five ethno-linguistically distinct endogamous tribal populations; Siddis and Gonds of Uttara Kannada district, Karnataka; Varli and Kolgha of Valsad district, Gujarat; and Dangi Konkana of Dang district, Gujarat. The genotype data obtained after molecular analysis of the three DRD2 sites was subjected to statistical analysis such as calculation of allele frequencies, haplotype frequencies among others. Subsequently, a neighbor-joining tree was also constructed from the data obtained.

**RESULTS:**

The three DRD2 sites were found to be polymorphic in all the populations. All the populations showed high levels of heterozygosities. Out of the eight possible haplotypes, most populations shared seven haplotypes. Of all the populations, Siddis showed the highest frequency of the ancestral haplotype B2D2A1 (11.4%). Significant LD was found to exist for *TaqI* ‘A’ and *TaqI* ‘B’ sites in both the populations.

**CONCLUSION:**

The findings are in concurrence with those from other Indian studies, especially from Dravidian-speaking South Indian populations. Similar pattern of diversity observed for ethnically and linguistically diverse populations in the present study is indicative of complex structure of Indian populations.

## Introduction

Over the years, population genetic studies have seen massive progression from the use of unlinked markers to linked markers. This has enabled haplotype construction to study population dynamics, which further provides meaningful insights both in the evolutionary and disease association studies. The global survey of variation in the dopamine receptor D2 (*DRD2*) system, located on chromosome 11,[1] has brought into focus the significance of the gene while studying genetic structure of human populations using haplotype analysis.[2] Thus, in the present study, *DRD2* locus has been used to understand the biologic consequences in terms of the population structure, among the five endogamous population groups with distinct ethnic and linguistic backgrounds, chosen from South Indian state of Karnataka and the West Indian state of Gujarat.

## Materials and Methods

This study was undertaken with an aim to get an understanding of the distribution pattern of *DRD2* allele and haplotype frequencies in five culturally distinct populations in light of their ethno-historical accounts. The description of the study populations is as follows:

**Siddis:** Siddi is a tribal group in India with an African ancestry,[3–5] majorly distributed in the states of Karnataka and Gujarat. India experienced first migratory wave of the Siddis during the 12^th^ century. Ancestors of Siddis residing in interior areas of Karnataka were brought as slaves by the Portuguese to Goa in the 16^th^ and 17^th^ centuries. The Siddi population (10,500) inhabiting Uttara Kannada district is primarily distributed in six talukas, namely, Sirsi, Mundagoda, Joida, Haliyala, Yellapura, and Ankola.[6] In their somatoscopic features (broad nose, dark skin color, kinky hair, alveolar prognathism, etc.), the Siddis resemble Africans even today. The Siddis also show sign of the clan system that once existed among the Siddis of Africa.[6] Ethno-historical records have revealed a long period of Portuguese and South Indian contact with the Siddis, and therefore, biological affinities of the Siddis with them cannot be ruled out. The social and cultural ties of the Siddis to their immediate neighbors are so strong that they have become bilingual and speak Konkani, a language belonging to the Indo-European family of languages, and Kannada, a language belonging to the Dravidian group of languages, and have abandoned the Swahili family of languages to which they originally belonged.

**Gonds:** Gonds is the largest tribal group of India, concentrated in Madhya Pradesh, eastern Maharashtra, Chhatisgarh, northern Andhra Pradesh, western Orissa and northern Karnataka. The study samples were collected from Bhatkal taluk of Uttara Kannada district of Karnataka. They are considered to have migrated here from Adilabad district of Andhra Pradesh in 18^th^ century. Gonds in Karnataka number about 8000[7] and contribute to 1.33% of the total Gond population in India. The group speaks Kannada, a language belonging to Dravidian linguistic family. As Bhatkal is situated near the Karnataka-Goa border, Konkani language finds its place in a few words during conversation. They follow clan exogamy and community endogamy. The Gonds exhibit physical features of that of the ‘Dravidians’, who are characterized by medium stature, brownish black skin, dolicho-cephalic head and mesorrhine nose. The hair is plentiful which is wavy with an occasional tendency to curl.

**Varli:** The term Varli has been derived from the Sanskrit word ‘Varal’ meaning uplander. They are generally found to inhabit hilly terrains. Varlis are famous for their ancient Indian folk art tradition of painting. Historians believe that the Varli tradition can be traced back to the Neolithic period between 2500 BC and 3000 BC, indicating the antiquity of the tribe. Varlis have migrated to South Gujarat from Konkan area of Maharashtra state. They are physically tall, dark, slim and well built, with features described as proto-Australoid. They were traditionally hunters and gatherers but now majority of the tribesmen own small land holdings. They have four endogamous divisions, namely, Shuddha, Murdes, Davars and Nihirs. Each of the four divisions has exogamous clans. Their population size in Gujarat is 255, 271.[7] Linguistically, they are affiliated to Indo-European language family.

**Dangi Konkana:** The Konkanas in Gujarat are immigrants from western coastal strip of Maharashtra, western India. They are dark, short-statured people and show ethnic affinities with Varlis. Konkanas largely depend upon agriculture, agriculture labor, fishing and collection of minor forest products for their subsistence. The community is divided into a number of exogamous units like Mahala, Gavid, Gavit, Gaikwad, etc. They practice group endogamy and clan exogamy. The Konkanas of district Dang are known as the Dangi Konkana. The total population of Dangi Konkana is 50,201.[7]

**Kolgha:** The Kolghas are classified as a primitive tribal group (PTG) in Gujarat state of India and their population size is 48,000.[7] Spoken dialect of Kolgha has a strong admixture of Indo-European and Dravidian language family words. They are mainly dependent on labor, cattle grazing and tanning of animal hides for their subsistence. Kolghas are divided into several exogamous clans.

The gene under study, *DRD2*, spans over 270 kb and has been mapped to locus 11q22.3-q23.1.[8] It encodes the D2 subtype of dopamine receptor which is one of the five types of dopamine receptors encoded by five separate genes. These receptors are known to mediate enzyme activities, metabolic rates, and ion channels and are involved in neurological signaling and functioning.[9] *DRD2* gene is of special interest as it is a target site of many neuropsychiatric drugs, and is thus of prime concern in the fields of neurology, psychiatry, endocrinology, among others. It is a strong candidate gene implicated in alcoholism and other substance use disorders.[10–12] Beginning with detection of *TaqI* ‘A’ site,[13] several other restriction polymorphism sites have been identified mostly in the non-coding region of this gene,[14] of which the *TaqI* ‘A’ site is the one most frequently studied in association studies.[13,15] The gene is being increasingly studied because of not only its functional significance but also its evolutionary significance. Three restriction site polymorphisms (RSPs) that are of special interest in finding out evolutionary relationships with reference to the *DRD2* locus are *TaqI* ‘A’, *TaqI* ‘B’ and *TaqI* ‘D’. These three *TaqI* restriction fragment length polymorphisms (RFLPs) are used to construct haplotypes in the order *TaqI* ‘B’, *TaqI* ‘D’ and *TaqI* ‘A’. The site-present alleles at the three sites are represented as ‘B2’, ‘D2’ and ‘A2,’ respectively, and the site-absent alleles are represented as ‘B1’, ‘D1’ and ‘A1,’ respectively, where B2, D2 and A1 alleles are the ancestral alleles.[2,15,16]

A total of 232 chromosomes were typed in the five populations for the three autosomal co-dominant biallelic *DRD2* sites – *TaqI* ‘A’, *TaqI* ‘B’ and *TaqI* ‘D’, but the reported data are for lesser number of chromosomes because of technical errors. Distribution of the samples by birth place and tribes is given in Table 1.

**Table 1:** Distribution of samples by tribes and birth place

Population	Birth place	Sample size (N)
Siddis	Uttara Kannada, Karnataka	48
Gonds	Uttara Kannada, Karnataka	46
Varli	Valsad, Gujarat	42
Dangi Konkana	Dang, Gujarat	46
Kolgha	Valsad, Gujarat	50
Total		232

The three sites, *TaqI* ‘A’,[17] *TaqI* ‘B’[18] *TaqI* ‘A,’[19] have been described previously. Five milliliters of intravenous blood was collected from individuals unrelated up to at least first cousin level by a trained medical practitioner after taking informed consent from them. Following blood collection, DNA was isolated using salting-out method.[20] The three *DRD2* sites were amplified using the standard primers and protocols.[15,21] The polymerase chain reaction (PCR) products were then digested with the restriction enzyme *TaqI* as per the manufacturer’s recommended conditions. Electrophoresis was subsequently carried out in 2% agarose gel stained with ethidium bromide for visualization. Following this, data obtained were subjected to statistical analysis.

All the procedures of data collection and analysis were in accordance with ethical standards of the Helsinki Declaration (1975).

## Results

Allele frequency estimates for the three *DRD2* sites in the five populations were made by direct gene counting and the assumption of Hardy-Weinberg Equilibrium was tested using χ^2^ goodness-of-fit test. The estimates are presented in Table 2.

**Table 2:** Ancestral allele frequencies at individual sites of *DRD2* locus and average heterozygosity

Population	*TaqI* ‘B’			*TaqI* ‘D’			*TaqI* ‘A’		
	B2	2n*	χ^2^†	D2	2n	χ^2^	A1	2n	χ^2^
Siddis	0.706	92	1.95	0.691	68	0.038	0.341	88	0.354
Gonds	0.693	88	0.655	0.608	74	0.048	0.258	66	0.544
Varli	0.679	84	1.379	0.695	82	0.020	0.333	84	2.634
Dangi Konkana	0.652	92	0.872	0.678	90	0.815	0.326	92	0.005
Kolgha	0.530	100	0.294	0.790	100	0.463	0.459	98	0.149

* 2n, number of chromosomes tested;

† χ^2^ values are nonsignificant at df = 1 and *P* < 0.05

All the three *TaqI* sites are found to be polymorphic in all the populations and none is found to show significant departure from Hardy-Weinberg Equilibrium at 5% level of significance. B2 and D2 alleles have frequencies greater than 60% in all the populations, the only exception being Kolgha which shows a frequency of 0.53 for the B2 allele. Kolgha shows highest frequency values for the ancestral alleles D2 (0.79) and A1 (0.459), whereas highest frequency for ancestral allele B2 (0.706) is seen in Siddis. Of the three sites, greater variation is seen in frequency distribution of the A1 allele.

Average heterozygosity was computed according to Nei[22] and the values are presented in Table 3.

**Table 3:** Average heterozygosity at *DRD2* locus

Population	Average heterozygosity
Siddis	0.4359
Gonds	0.4341
Varli	0.4405
Dangi Konkana	0.4482
Kolgha	0.4467

All the study populations show high level of diversity with respect to the three sites and the heterozygosity values range from 0.4341 among Gonds to 0.4482 among Dangi Konkana.

Within each population, haplotype frequencies were estimated by maximum likelihood method from the multi-site marker typing data, using the program HAPLOPOP. The haplotype frequencies for the five populations are presented in Table 4.

**Table 4:** Haplotype frequencies at *DRD2* locus

Haplotype	Siddis	Gonds	Varli	Dangi Konkana	Kolgha
B1-D1-A1	0.000	0.000	0.000	0.000	0.000
B1-D1-A2	0.000	0.050	0.000	0.017	0.000
B1-D2-A1	0.229	0.159	0.265	0.280	0.382
B1-D2-A2	0.052	0.051	0.065	0.050	0.077
B2-D1-A1	0.000	0.050	0.032	0.023	0.017
B2-D1-A2	0.312	0.289	0.273	0.286	0.197
B2-D2-A1	0.114*	0.051*	0.045*	0.023*	0.060*
B2-D2-A2	0.292	0.351	0.321	0.320	0.267

* Ancestral haplotype frequency

When haplotype diversity is taken into account, it is found that of the eight possible three-site haplotypes, no population shows all the eight haplotypes. Gonds and Dangi Konkana each show seven haplotypes; Varli and Kolgha each exhibit six haplotypes; and Siddis have only five haplotypes. All the populations lack B1D1A1 haplotype in common. The next most frequently absent haplotype is B1D1A2. The three haplotypes, namely, B2D2A2, B2D1A2 and B1D2A1, together occur with a frequency greater than 80% in all the study populations. The ancestral haplotype B2D2A1 is found to be present in highest frequency in Siddis (11.4%) and lowest in Dangi Konkana (2.3%).

The standardized pairwise linkage disequilibrium (LD) value D’ was computed for each pair of markers[23] Data on pairwise LD values for the three *DRD2* sites are shown in Table 5.

**Table 5:** Standardized pairwise LD values at *DRD2* locus

Population	*TaqI* ‘B’ and *TaqI* ‘D’ sites	*TaqI* ‘A’ and *TaqI* ‘D’ sites	*TaqI* ‘B’ and *TaqI* ‘A’ sites
Siddis	–0.094*	–0.114*	0.131*
Gonds	–0.034	0.043	0.081*
Varli	–0.100384*	–0.066707	0.152114*
Dangi Konkana	–0.095298*	–0.073578*	0.164014*
Kolgha	–0.098398*	–0.082060*	0.170880*

* Significant at df = 1 and *P* < 0.05

The values are generally low (<0.2) in all the comparisons. All the populations show significant values in the comparison between *TaqI* ‘B’ and *TaqI* ‘A’ sites. Except for Gonds, all other populations show significant LD between sites *TaqI* ‘B’ and *TaqI* ‘D’; three of five populations show significant values for LD between *TaqI* ‘A’ and *TaqI* ‘D’ sites.

**Figure 1:**
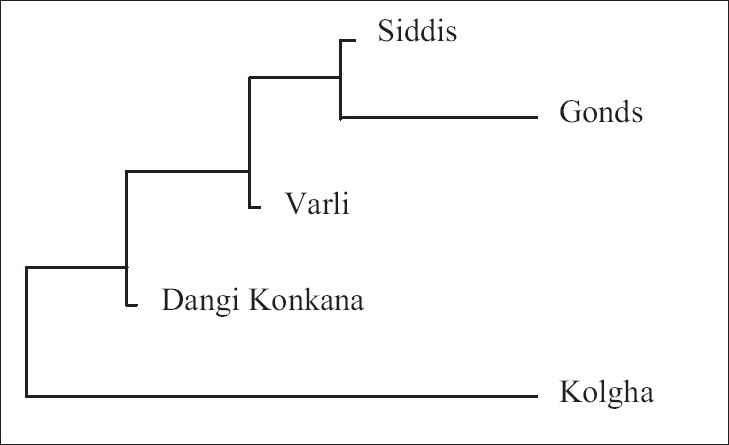
Neighbor-joining tree depicting genomic relationships among the study populations

A dendrogram was constructed using the neighbor-joining (NJ) method[24] to identify affinities among the study populations and is given in Figure 1.

Siddi population is grouping with Gonds on one hand and Kolgha is lying on the other extreme of the NJ tree and is closer to Dangi Konkana and Varli than the two South Indian populations.

## Discussion

The three *TaqI* sites define evolutionary relationships of the *DRD2* haplotypes. Alleles at the three sites show a variable pattern of distribution across different populations of the world or more so across different geographic areas. Ancestral allele B2 is observed at higher frequencies than B1 allele in African (>0.72) and European populations (>0.68) as compared to other world populations.[2,25,26] Ancestral allele D2 has higher frequency than D1 in all the world populations with the exception of European populations. On the other hand, ancestral allele A1 is found to have lower frequency than A2 in all world populations except in American populations.[2] In the Indian context it is seen that the frequency of ancestral allele B2 at *TaqI* ‘B’ site varies from 36.67% in Onge tribe[27] to 91% in Toda tribe.[25] It is observed that range is not a reliable parameter for comparison across populations because most populations show intermediate values; median values of the allele frequencies have thus been considered for comparing populations. It is seen that the median value computed for the B2 allele (0.679) in the present study is close to its corresponding value of 0.68 computed from various available studies on Dravidian-speaking tribal populations of South India,[25,27–29] but not close to the median value of 0.9 and 0.85 obtained for African and European populations, respectively,[2] and the median value of 0.81 computed for Indo-European speaking North Indian population groups.[30] Similar observations have been made for D2 allele. Thus, the findings from the present study are broadly in concurrence with the findings from Dravidian-speaking South Indian populations than other studied populations. Except for Kolgha, all populations are showing comparable values of ancestral alleles at the three loci. The estimated levels of heterozygosities are high in all the study populations, comparable to the corresponding findings from other Indian studies.[25,27–29]

As also seen in other studies,[25,27,28] significant disequilibrium is observed among the *TaqI* ‘A’ and *TaqI* ‘B’ Single Nucleotide Polymorphism (SNPs) in all the study populations, although not as high.

Haplotype distribution pattern reveals haplotype sharing between the study populations and a similar pattern of distribution. Most of the study populations are showing ancestral haplotype B2D2A1 at a frequency >5%. This is in contrast to the observation made by Kidd *et al*.[2] that the ancestral haplotype is common only in Africa but is rare or absent elsewhere. Siddi especially is showing the ancestral haplotype in appreciable frequency (11.4%). This frequency is higher than that observed in most Indian studies,[25,28] but lower than that found for Siddis of Gujarat[27] and Thotis and Nayakpods of Andhra Pradesh.[29] The values observed are comparable to findings from other South Indian studies[25,27–29] The recently derived haplotypes B1D1A1 and B1D1A2 are either absent or present at low frequencies in the study populations.

Structure of any population is affected by various factors such as demographic history, size, ethnicity, drift, differential selection, among others. The population groups in the present study are ethnically, geographically and linguistically distinct. Except for Kolgha (which is a PTG), all other study groups are immigrants to their current geographic location. Siddis are African immigrants, who were brought to west coast of India by Portuguese in the 16^th^ and 17^th^ centuries.[7] Similarly, Gonds of Uttara Kannada district of Karnataka have migrated from Adilabad district of Andhra Pradesh in 18^th^ century,[31] and since then have lost marital alliance with all members of parental group. Dangi Konkana and Varlis have migrated from Konkan coast of Maharashtra to Gujarat. Despite their diverse ethnic backgrounds and linguistic affinities, there seems to be an underlying genetic uniformity in these groups with respect to the *DRD2* locus as evident from allelic and haplotype distribution pattern. These similarities could either be because of their common ancestor stock and/or a result of long ethno-historical contacts that might have shaped their biologic structure also. It is clear from the NJ tree that the Siddi group, having African ancestry, is separated from other groups but closer to its geographic neighbor. Also, Kolgha, a PTG, is positioned separately from other groups but is closer to its geographic neighbors. Although only one locus has been considered in the present study, haplotype analysis is known to be a robust method for studying population structure. Thus, the results obtained in the present study indicate that the structure of Indian populations is complex and is the by product of cultural, temporal and spatial changes over a period of time.
